# Bioinspired pressure-sensitive adhesive: evaluation of the effect of dopamine methacrylamide comonomer as a general property modifier using molecular dynamics simulation

**DOI:** 10.1039/d1ra03634c

**Published:** 2021-06-08

**Authors:** Mahmoud Heydari, Farhad Sharif, Morteza Ebrahimi

**Affiliations:** Department of Polymer Engineering and Color Technology, Amirkabir University of Technology Tehran 15875-4413 Iran Sharif@aut.ac.ir

## Abstract

The use of catechol-containing comonomers as a general property enhancer to achieve unique properties has received particular attention for designing bioinspired polymeric materials. In this study, molecular dynamics simulation was used to investigate the role of dopamine methacrylamide (DMA) and *N*-phenethyl methacrylamide (PMA) comonomers in chain conformation and their effects on the mechanical properties and adhesion of poly(*n*-butyl acrylate-*co*-acrylic acid) copolymer. Addition of 4% by weight of DMA comonomer in the terpolymer structure reduces the gyration radius of the poly(*n*-butyl acrylate-*co*-acrylic acid) copolymer. This reduction is due to the formation of intramolecular hydrogen bond interactions. A further increase in the DMA up to 12.2% by weight increases the radius of gyration by 5%. The effect of PMA on the gyration radius of the poly(*n*-butyl acrylate-*co*-acrylic acid) copolymer is more extensive, compared to DMA. While DMA enhances both van der Waals and electrostatic components of the cohesive energy density through increasing π–π interactions and hydrogen bond formations, PMA only improves the van der Waals component. Assessment of mechanical properties revealed that the addition of DMA comonomer resulted in a transition from brittle to tough behavior in poly(*n*-butyl acrylate-*co*-acrylic acid) pressure-sensitive adhesive. Ductility index improvement by DMA is higher than that by PMA. DMA comonomers accumulate on the silica surface resulting in the terpolymer chains approaching the dry silica surface from the hydroxyl groups of the catechol. Accumulation of DMA only enhances the cohesive energy and does not improve the adhesive energy.

## Abstract 2

The use of catechol-containing comonomers as a general property enhancer to achieve unique properties has received particular attention for designing bioinspired polymeric materials.

## Graphical abstract

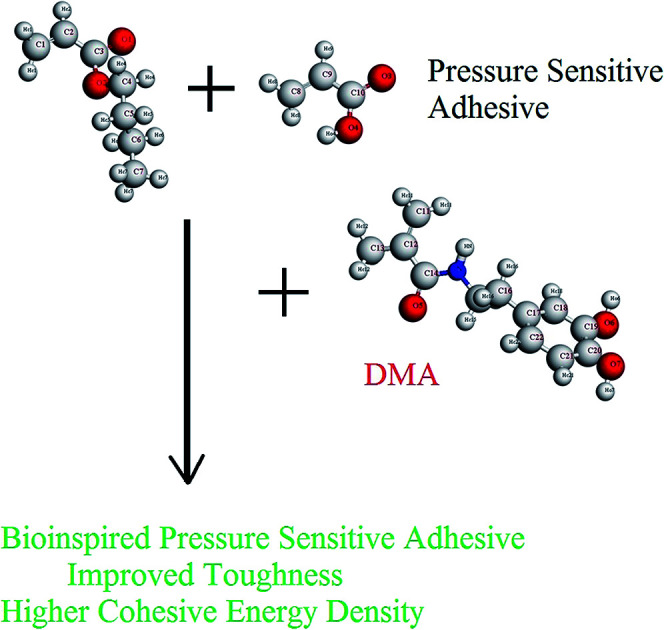

## Introduction

The use of catecholic amino acid as a property modifier for the production of high performance products has received special attention recently. Dopamine methacrylamide (DMA) containing 3–27% mussel foot proteins consists of amino acid and catechol groups. The special properties of the DMA are due to its catechol group containing a couple of hydroxyl groups attached to a benzene ring. This group provides the ability to interact with a variety of surfaces. These interactions include hydrogen bond formation, π–π interaction, and the formation of covalent and coordination bonds as chemical bonds.^1^

DMA can be used in designing and manufacturing some products with advanced technology such as membranes,^2,3^ antibacterial nano-particles,^4^ bioinspired hydro-gels with high memory capacity,^5^ dielectric materials with strong energy storage capability,^6^ tissue engineering,^7^ and adhesives with special properties.^8^

Yang *et al.*^9^ found that the radical polymerization of 5% by weight of DMA with 2-methoxy ethyl methacrylate significantly improved the adhesion properties. This percentage of DMA was the optimal percentage for both the adhesion strength and the contact angle with the substrate surface. In addition, Lee *et al.*^10^ showed that the use of this copolymer as a primer increased the adhesion of commercial dental adhesives to the tooth surface. Payra *et al.*^11^ investigated the effect of different percentages of DMA on alkyl methacrylates with various alkyl chain lengths. They found that DMA improved the mechanical properties of branched alkyl methacrylates more than linear alkyls did. Furthermore, they showed that small amounts of DMA in the structure of alkyl methacrylates significantly improved the corrosion resistance.^12^

Glass *et al.*^13^ found that coating the filament surface with poly(dopamine methacrylate-*co*-methoxyethyl acrylate) copolymer significantly enhanced its adhesion to the studied surfaces. DMA improved adhesion more in the wet environment compared to the dry condition. Tiu *et al.*^14^ reported that DMA comonomer improved the adhesion strength of poly(*n*-butyl acrylate-*co*-acrylic acid) copolymer to different organic and metal surfaces. Furthermore, they showed^15^ that a combination of catechol and amino acid in a side group led to the highest improvement in the performance of the adhesive based on copolymers of poly(*n*-butyl acrylate-*co*-acrylic acid) in the peeling, static shear and atomic force spectroscopy tests. As a different approach, effect of hydroxyl and hydrogen group groups on the adhesion of liquid to substrate have been discussed in literature^16,17^ by using fluorosilanes on the substrate.

Molecular dynamics simulation is a powerful method for calculation of the behavior of polymers in an equilibrium state and prediction of various properties. This tool can be used to understand and determine the molecular mechanism and to predict some macro properties such as mechanical,^18–22^ rheological characteristics,^23,24^ drug delivery,^25,26^ and adhesion.^27–31^ The role of comonomers and functional groups,^32^ surface modification,^33^ surface topology,^34^ additives, and small molecules^35^ in the adhesion of polymers to different surfaces can be determined using molecular dynamics simulation.

Despite the increasing use of DMA in the design and synthesis of various types of bioinspired adhesives, the literature lacks a comprehensive study about the mechanism of the improvement of various properties using molecular dynamics simulation method. In general, the explanations and mechanisms presented in the literature about the reasons for promoting adhesion, cohesion, and mechanical properties observed in experimental data have not been proven at the atomistic scale. It is very difficult and almost impossible to evaluate these mechanisms in microscopic and atomic scales by experimental methods. The present study aimed to explore improvement mechanisms and to find answers about the impact of DMA comonomer on inter-chain and intra-chain interactions at the atomistic scale using the molecular dynamics method. Studying these mechanisms helps to design new adhesives using synthesis copolymers or surface modification of substrates. In the present study, the effect of DMA comonomer on the gyration radius, cohesive energy density, hydrogen bond formation and mechanical properties of poly(*n*-butyl acrylate-*co*-acrylic acid) copolymer was investigated using the molecular dynamics simulation. For a more detailed study of the role of the catechol group, a control sample of *N*-phenethyl methacrylamide (PMA) was considered simultaneously. The difference between these two comonomers is in the hydroxyl groups on the benzene ring of DMA. Then, the interaction of different functional groups of terpolymer containing DMA or PMA comonomer with the surface of a dry silica layer was investigated using molecular dynamics simulation to determine the ordering, closeness, and affinity of various functional groups of the terpolymers to the surface.

## Methods

Table 1 presents the names of copolymers and terpolymers abbreviated according to the comonomer composition used in their structure. The chain length was considered 40 for the copolymer and all terpolymers.

**Table 1:** Names and comonomer contents of copolymer and terpolymers

Name	Number of butyl acrylate monomers	Number of acrylic acid monomers	Number of dopamine methacrylamide monomers	Number of *N*-phenethyl methacrylamide monomers	Number of chains
36-4	36	4	0	0	15
DMA 35-4-1	35	4	1	0	15
DMA 34-4-2	34	4	2	0	15
DMA 33-4-3	33	4	3	0	15
PMA 34-4-2	34	4	0	2	15

All simulation steps were performed using the Materials Studio software version 4.3. According to a review of the literature, the COMPASS (condensed-phase optimized molecular potentials for atomistic simulation studies) force field was used in this study.^36–40^ The single chain energy of poly(*n*-butyl acrylate-*co*-acrylic acid) copolymer, poly(*n*-butyl acrylate-acrylic acid-dopamine methacrylamide), and poly(*n*-butyl acrylate-acrylic acid-phenethyl methacrylamide) terpolymers was minimized with a force convergence threshold and energy convergence threshold of 0.005 kcal mol^−1^ Å^−1^ and 0.0001 kcal mol^−1^, respectively.^41,42^ Subsequently, 15 copolymer or terpolymer chains were placed in simulation boxes with an initial density of 0.5. For coulombic interactions, the Ewald summation method was applied with an accuracy of 0.001 kcal mol^−1^.^41^ An atom base method was used with a cut-off distance of 12.5 angstroms for van der Waals interactions.^42^ Again, simulation box relaxation was done using the smart algorithm with an energy convergence threshold of 0.0001 kcal mol^−1^ and a force convergence of 0.005 kcal mol^−1^ Å^−1^. The minimized simulation boxes reached equilibrium density using the NPT ensemble in 1 ns. Then, to remove any residual stress, the systems were annealed twice in the NVT ensemble through heating from 298 K to 548 K and cooling back to 298 K with temperature and time intervals of 50 K and 100 ns, respectively. In other words, the entire annealing time was 2400 ps.

Annealing was performed from 298 K, as the ambient temperature, to 548 K, which is sufficiently higher than the glass transition temperature of copolymer and terpolymers,^43^ to ensure system relaxation. A review of the literature indicated that a temperature interval of 50 K is appropriate for our system.^41,42^ Moreover, the temperature interval of 50 K was selected for annealing steps to reduce computational time and cost. The temperature and potential energy fluctuations of copolymer and terpolymers in each step of annealing demonstrated that 100 ns is a sufficient time for the system to reach the equilibrium condition.

Finally, the systems were equilibrated again for 2 ns at 298 K. In order to ensure the equilibrium state, the method proposed by Liu *et al.*^44^ was evaluated for the studied systems, which states that temperature and energy fluctuations less than 5% around a constant value confirm an equilibrium condition. Other researchers have also used this method for evaluating the equilibrium condition.^45,46^

**Fig. 1:**
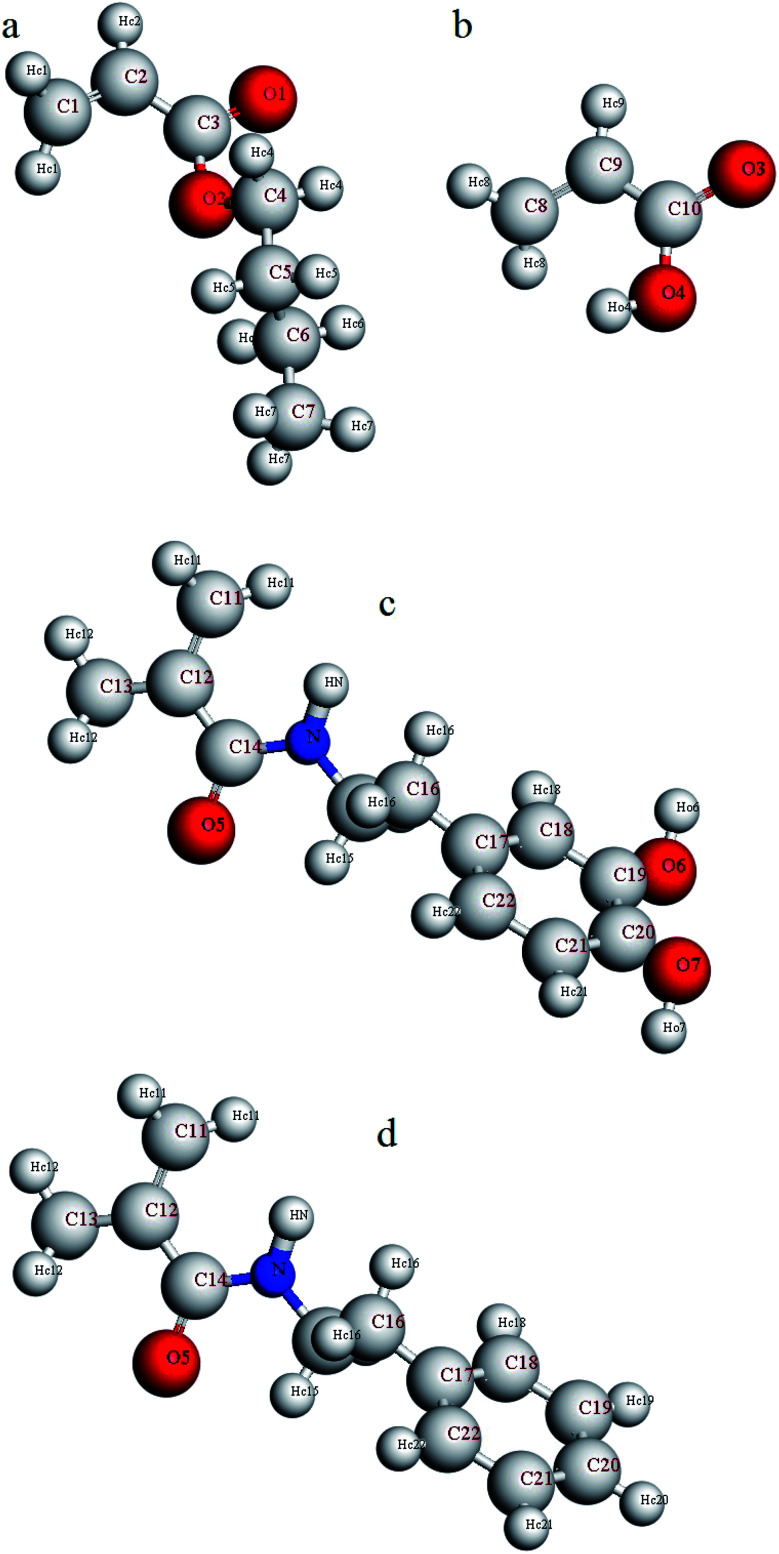
Naming the atoms of comonomers used in the structure of copolymer and terpolymers (a) butyl acrylate, (b) acrylic acid, (c) dopamine methacrylamide, and (d) *N*-phenethyl methacrylamide.

Furthermore, in order to investigate adhesion to the silica surface, a layer of copolymer or terpolymer was placed on a silica layer (dimensions: *a* = 54.052, *b* = 51.057, *c* = 16.461). The silicon and oxygen atoms of the silica layer were saturated with hydroxyl and hydrogen groups, respectively.^47^ A vacuum layer with a thickness of 100 angstroms was considered on the copolymer or terpolymer layer to build the final simulation box. This vacuum layer was considered to limit interactions with the upper atoms of the silica layer and to increase the computing speed.^48^ Considering the silica layer as a fixed and rigid layer, the minimization of the final simulation box was performed using the smart method. Again, the relaxed simulation box was annealed twice from 298 K to 598 K and back to 298 K in the NVT ensemble. Finally, it was equilibrated in the NVT ensemble for 2 ns. For a more detailed analysis of the simulation results, the atoms of comonomers used in the copolymer and the terpolymers were named according to Fig. 1. Hydrogen atoms were named according to the bonded atom as subtitles. For example, H_O4_ means a hydrogen atom bonded to O4 oxygen atoms or H_N_ means a hydrogen atom bonded to N atom.

## Results and discussion

The presence of DMA and PMA comonomers led to extensive changes in the conformation, internal energy, mechanical properties, adhesion, and cohesion of poly(*n*-butyl acrylate-*co*-acrylic acid) copolymer as below.

### Gyration radius

The equilibrium radius of gyration is a measure of the size of the polymer chain in the equilibrium state and is equivalent to the square root of the distance between the segments of the polymer chain and its center of mass.1
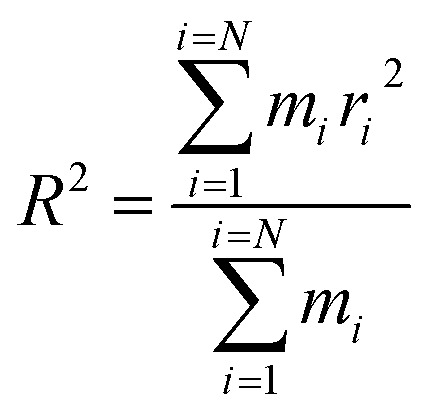
In this equation, *R*, *r*_*i*_, and *N* are the radius of gyration, distance of segment *i* from the center of mass, and number of segments, respectively.

**Fig. 2:**
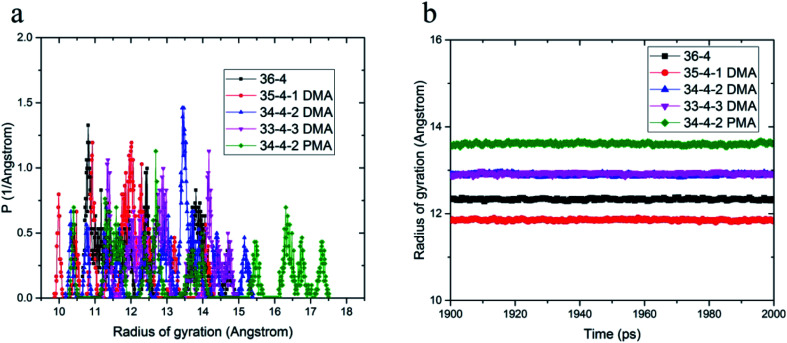
(a) Distribution of gyration radius and (b) average gyration radius in equilibrium state of copolymers and terpolymers.

The effect of different compositions of DMA and PMA comonomers on the distribution of gyration radius and average gyration radius of the terpolymers in the equilibrium state are shown in Fig. 2. PMA 34-4-2 terpolymer containing PMA comonomer had the largest radius of gyration compared to other terpolymers containing DMA comonomers.

Initially, the presence of one unit of DMA comonomer in the terpolymer chain, equal to 4.3% by weight, reduced the gyration radius of DMA 35-4-1 terpolymer compared to 36-4 copolymer. The hydroxyl groups on the benzene ring could increase the intramolecular interaction by hydrogen bond formation, resulting in a reduction in the size of chains and the gyration radius. However, the gyration radius increased with an increase in the DMA content in the chains up to 2 and 3 units, equal to 8.7% and 12.82% by weight, in DMA 34-4-2 and DMA 33-4-3 terpolymers compared to 36-4 copolymer. The large side group of DMA containing a benzene ring creating steric effects causes the polymer chains to expand in space in DMA 34-4-2 and DMA 33-4-3 terpolymers. PMA comonomer increased the gyration radius more than DMA because of the absence of hydroxyl groups on the benzene ring of PMA. As mentioned earlier, intramolecular hydrogen bond formation by hydroxyl groups on DMA diminished the chain expansion caused by the steric effects of large side groups.

A larger radius of gyration and a greater chain expansion mean a closer contact between chains, which improves intermolecular interactions. To investigate this issue, the cohesive energy densities of the terpolymers were compared with 36-4 copolymer.

### Cohesive energy density

**Fig. 3:**
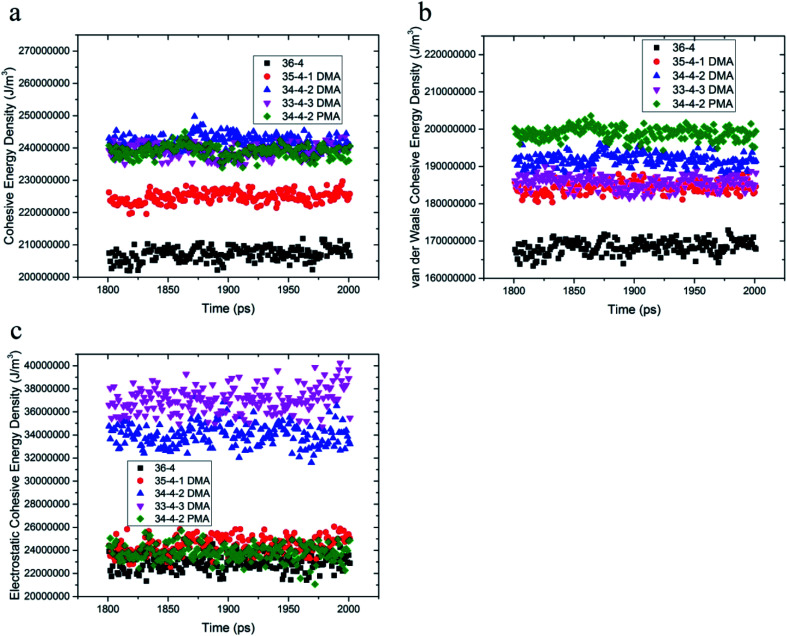
(a) Total cohesive energy density, (b) van der Waals, and (c) electrostatic components.

Cohesive energy density is a measure of intermolecular interactions and is equal to pulling out a molecule from its neighboring interactions. DMA and PMA comonomers increased π–π and hydrogen bond interactions between the chains, which increased the van der Waals and electrostatic interactions, respectively. The total cohesive energy density and van der Waals and electrostatic components of the copolymer and terpolymers are shown in Fig. 3. According to Fig. 3a and b, the van der Waals component of the cohesive energy density of 36-4 copolymer and other terpolymers was much larger than the electrostatic component confirming the main role of van der Waals interactions among the chains. DMA and PMA comonomers increased the cohesive energy density of poly(*n*-butyl acrylate-*co*-acrylic acid) copolymer. As shown in Fig. 3a, the increase in the total cohesive energy due to the presence of PMA in PMA 34-4-2 terpolymer compared to 36-4 copolymer was about 17%, which was approximately equal to the increase in the total cohesive energy in DMA 33-4-3 and DMA 34-4-2 terpolymers due to the presence of DMA. Fig. 3b shows that DMA and PMA comonomers significantly improved the van der Waals interaction between the chains. The increase in van der Waals interactions by PMA in PMA 34-4-2 terpolymer was greater than the increase by DMA comonomers in DMA 34-4-2 and DMA 33-4-3 terpolymers. As mentioned earlier, the larger gyration radius caused by PMA compared to DMA comonomers led to chain expansion and more contact between the terpolymer chains creating greater van der Waals interactions.

The similarity of the gyration radius of DMA 34-4-2 and DMA 33-4-3 terpolymers caused a similar increase in their van der Waals component of the cohesive energy density relative to 36-4 copolymer. Fig. 3c shows that PMA comonomer caused a negligible improvement in the electrostatic component of the cohesive energy density of 36-4 copolymer. However, changes in the electrostatic component of the cohesive energy density in DMA containing terpolymers were quite remarkable in DMA 34-4-2 and DMA 33-4-3. Comparison of Fig. 3b and c reveals that both van der Waals and electrostatic components were improved by DMA comonomer. Fig. 3c demonstrates that the role of electrostatic interactions in the total cohesive energy density became more pronounced upon increasing the percentage of DMA. The catechol groups, with the ability to enhance π–π and hydrogen bonds, act like a physical crosslink and increase the cohesive energy density. Improvement in the cohesive strength of poly(*n*-butyl acrylate-*co*-acrylic acid) with DMA commoner was reported by Tiu *et al.*^14^ using the static shear test. These authors stated that this enhancement could be due to hydrogen bond formation between catechols, as well as the π-stacking interactions of the DMA aromatic groups. The MD results of the present investigation supported their explanations.

### Hydrogen bonds

Hydrogen atoms, which bond to oxygen, nitrogen, fluorine, and chlorine by covalent bonds, can form hydrogen bonds with the mentioned atoms. The total number of hydrogen bonds of the copolymer and terpolymers are shown in Table 2.

**Table 2:** Number of hydrogen bonds of copolymer and terpolymers

Name	Number of hydrogen bonds
36-4	62
DMA 35-4-1	115
DMA 34-4-2	175
DMA 33-4-3	217
PMA 34-4-2	58

According to Table 2, DMA comonomer significantly increased the number of hydrogen bonds in the simulation box from 62 in 36-4 copolymer to 217 in DMA 33-4-3 terpolymer. However, comparison of the number of hydrogen bonds of 36-4 copolymer with PMA 34-4-2 terpolymer indicated that PMA comonomer had no effect on hydrogen bond formation. Radial distribution functions between oxygen and hydrogen atoms with the potential to form hydrogen bonds were evaluated to study the details of the hydrogen bonds formed by DMA comonomer. The radial distribution function *g*_A–B_(*r*), which is a measure of finding particle B relative to a reference particle A at a distance of *r*, is calculated as the ratio of the number of particles *n*_B_ in a spherical element with a thickness of d*r* to the total number of *N*_B_ that are in a cell with a total volume of *V* as follow:2
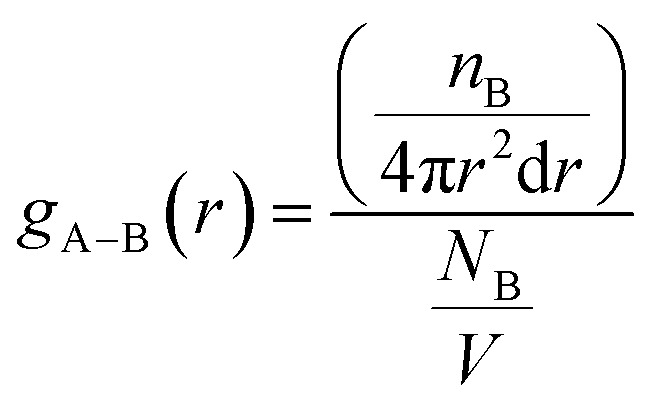


**Fig. 4:**
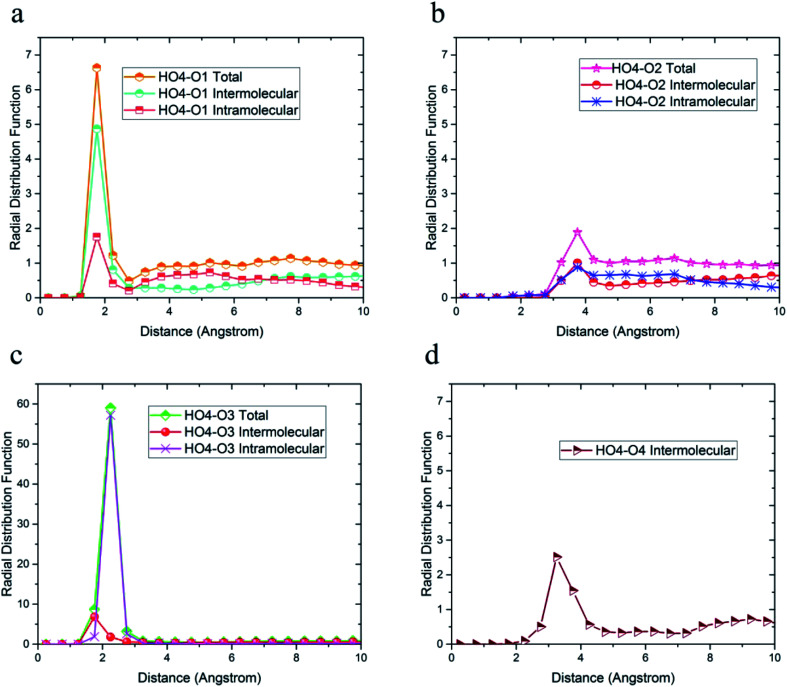
Radial distribution functions between H_O4_ hydrogen atoms and (a) O1, (b) O2, (c) O3, and (d) O4 oxygen atoms of 36-4 copolymer with intramolecular and the intermolecular components.

**Fig. 5:**
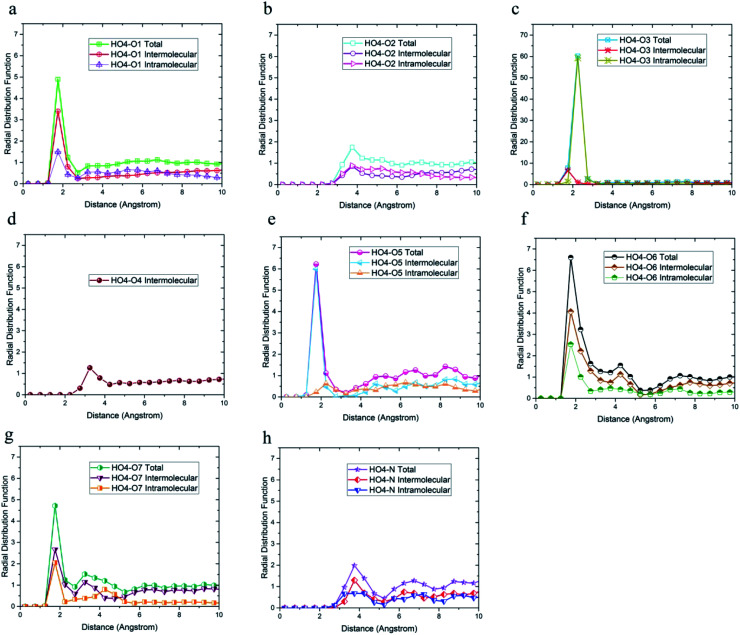
Radial distribution functions between H_O4_ and (a) O1, (b) O2, (c) O3, (d) O4, (e) O5, (f) O6, (g) O7, and (h) N atoms of DMA 33-4-3 terpolymer with intramolecular and the intermolecular components.

**Fig. 6:**
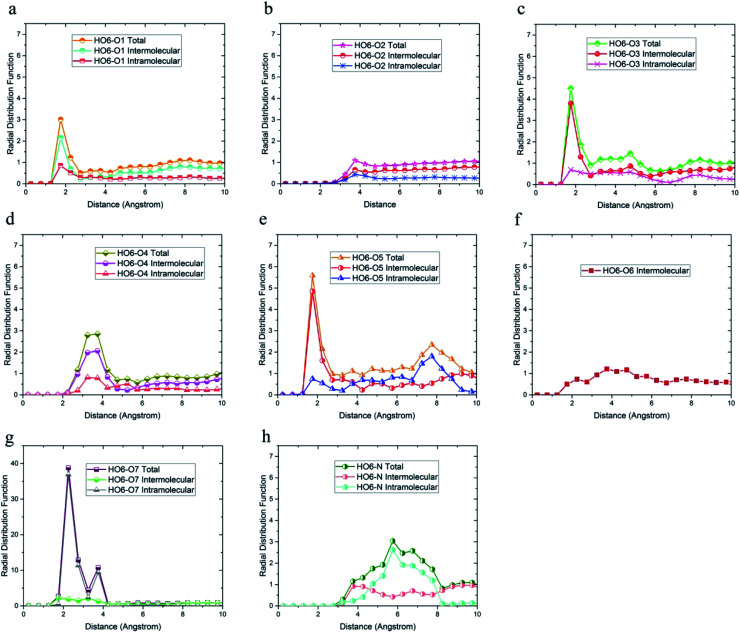
Radial distribution function between H_O6_ hydrogen atoms and (a) O1, (b) O2, (c) O3, (d) O4, (e) O5, (f) O6, (g) O7, (h) N of DMA 33-4-3 terpolymer with intramolecular and intermolecular components.

Due to the similarity of the results and to avoid any confusion, only the radial distribution functions between the oxygen and hydrogen atoms of 36-4 copolymer and DMA 33-4-3 terpolymer are shown in Fig. 4–6.

The radial distribution functions between H_O4_ hydrogen of 36-4 copolymer and all other oxygen atoms in the simulation box, *i.e.* O1, O2, O3, and O4 atoms, with intramolecular and intermolecular components are shown in Fig. 4. In general, peaks less than 3 angstroms are related to hydrogen bonds and chemical bonds, and peaks larger than this distance are related to van der Waals interactions.^41,49^ Thus, H_O4_ atoms could form hydrogen bonds with O1, O3, and O4 oxygen atoms as shown in Fig. 4. According to Fig. 4b, the peak in radial distribution function between H_O4_ and O2 was located at a distance of more than 3 angstroms, thus hydrogen bond formation was not possible. The inability to form a hydrogen bond with O2 is attributed to the steric effect of the side group and the lower partial charge of O2 compared to other oxygen atoms. Furthermore, the highest probability of the intermolecular hydrogen bond formation was observed between H_O4_ and O3 and O1 oxygen atoms, respectively.

The radial distribution function between H_O4_, H_O6_, H_O7,_ and H_N_ hydrogen atoms and oxygen and nitrogen atoms in the simulation box was considered to evaluate the effect of DMA on hydrogen bond formation in the DMA 33-4-3 terpolymer. According to Fig. 5, H_O4_ of DMA 33-4-3 terpolymer could form hydrogen bonds with O1, O3, O4, O5, O6 and O7 oxygen atoms because of the presence of the peak in a distance less than 3 angstroms.

In addition, the probability of intermolecular hydrogen bonds between H_O4_ and O5 was higher than other oxygen atoms. After that, the probability of forming intermolecular hydrogen bonds between H_O4_ and O6 was higher compared to other oxygen atoms. Furthermore, O3, O6, and O7 atoms were most likely to form intramolecular hydrogen bond with H_O4_. On the other hand, according to Fig. 5b and h, the peak was located at a distance of more than 3 angstroms indicating that hydrogen bonds were not formed with O2 and N atoms. In addition, the radial distribution function between H_O6_ and the other oxygen and nitrogen atoms of DMA 33-4-3 are shown in Fig. 6. Due to the similarity of H_O6_ and H_O7_ results, only the radial distribution functions between H_O6_ hydrogen and other oxygen atoms in the simulation are shown in Fig. 6. There was the possibility of hydrogen bond formation between H_O6_ hydrogen atoms and O1, O3, O4, O5, O6, and O7 atoms, while hydrogen bond formations with O2 and N atoms was not possible due to the steric effects.

**Fig. 7:**
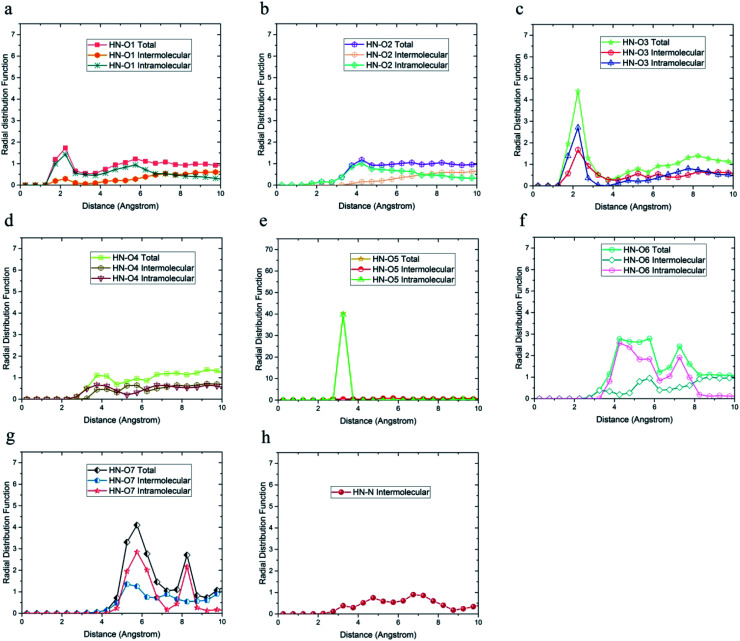
Radial distribution function between H_N_ hydrogen atoms and (a) O1, (b) O2, (c) O3, (d) O4, (e) O5, (f) O6, (g) O7, (h) N of DMA 33-4-3 terpolymer with intramolecular and intermolecular components.

As shown in Fig. 6, the highest probability of the intermolecular hydrogen bond formation was related to the O5 and O3 atoms. Furthermore, the probability of intramolecular hydrogen bonds between H_O6_ and O7 was higher compared to other oxygen atoms. The radial distribution functions between H_N_ and oxygen and nitrogen atoms of DMA 33-4-3 terpolymer are shown in Fig. 7.

As shown in Fig. 7, only in the radial distribution function between H_N_ and O1 and O3 atoms, the peaks were located at a distance of less than 3 angstroms; thus, only hydrogen bond formation with O1 and O3 oxygen was possible. As a result from Fig. 5–7, O5, O6, O7, N, H_O6_, H_O7_, and H_N_ atoms of DMA comonomer could participate in the formation of hydrogen bonds. Our simulation findings confirmed and clarified the mechanism of hydrogen bonds formation for cohesive strength improvement resulting from DMA comonomer.^14,15^

### Mechanical properties

Three methods including (1) static, (2) fluctuation formula, and (3) dynamics are used to calculate mechanical properties using molecular dynamics results.^45^ In this study, the static procedure was used to determine mechanical properties. The virial equation was used to estimate the tensor components. Due to the static condition, the first term of the right side of the below equation was removed.3
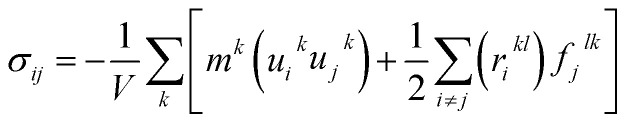
where *V*, *m*^*k*^, *u*^*k*^, and *r*^*kl*^ are the volume of the system, mass of particle *k*, velocity of particle *k*, and distance between particle *k* and *l*, respectively. In addition, *f*^*lk*^ is the force exerted from particle *k* to particle *l*. The elastic constant can be calculated by the first derivative of stress relative to strain. The shear and compressive modulus can be calculated from elastic constants using Voigt, Reuss, and Hill approximation according to the following equations.4
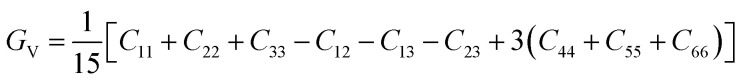
5
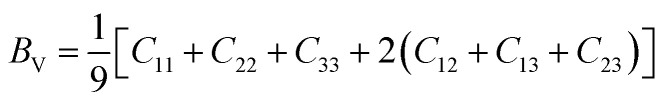

6 *G*_R_ = 15[4(*S*_11_ + *S*_22_ + *S*_33_ − *S*_12_ − *S*_13_ − *S*_23_) + 3(*S*_44_ + *S*_55_ + *S*_66_)]^−1^

7 *B*_R_ = [*S*_11_ + *S*_22_ + *S*_33_ + 2(*S*_12_ + *S*_13_ + *S*_23_)]^−1^

8
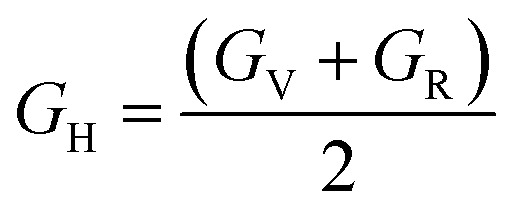
9
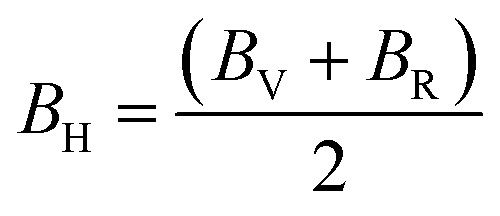
where *C* and *S* are the members of elastic modulus tensor and the compliance tensor, respectively. *G*_V_, *G*_R_, and *G*_H_ are shear moduli calculated using Voigt, Reuss, and Hill approximations, respectively. *B*_V_, *B*_R_, and *B*_H_ are compression moduli derived from Voigt, Reuss, and Hill approximations, respectively. Table 3 shows the results for the copolymer and terpolymers.

**Table 3:** Shear modulus, bulk modulus and ductility index of copolymer and terpolymers according to Reuss, Voigt and Hill approximations

Name	Model						
	Shear modulus			Bulk modulus			Ductility index
	Reuss	Voigt	Hill	Reuss	Voigt	Hill	*C*_12_ − *C*_44_
36-4	1.61	1.66	1.63	2.66	2.69	2.67	−0.487
DMA 35-4-1	1.68	1.78	1.73	2.93	3.25	3.09	0.478
DMA 34-4-2	1.53	1.77	1.65	2.89	2.91	2.90	0.821
DMA 33-4-3	1.42	1.58	1.50	3.39	3.51	3.45	0.975
PMA 34-4-2	1.68	1.75	1.72	2.91	2.94	2.93	0.279

According to Table 3, increasing the number of DMA comonomers reduced the shear modulus and increased the compressive modulus. *C*_12_ − *C*_44_ is the ductility index as a measure of the system toughness.^42^ The negative and positive values of ductility index indicate the brittleness and toughness behavior, respectively. A greater positive value of the ductility index means more toughness. According to Table 3, 36-4 copolymer was brittle, while the terpolymer containing DMA and PMA comonomers showed transition from brittle to ductile behavior. Comparison of the ductility index of PMA 34-4-2 and DMA 34-4-2 terpolymers demonstrated that DMA comonomer had a greater effect on toughness enhancement compared to PMA. Toughness improvement causes the adhesive to deform as plastic and to stretch longer leading to a more uniform distribution of the applied stress. On the other hand, the brittle behavior increases the stress concentration and crack growth under the applied stress. Therefore, DMA comonomer had a larger effect on improving the mechanical behavior of poly(*n*-butyl acrylate-*co*-acrylic acid) copolymer compared to PMA, which is in line with the results of a study by Meredith *et al.*^50^ Furthermore, Payra *et al.*^11^ showed that the toughness of poly(ethylhexyl methacrylate) increased continuously up to 8 mol% of DMA. However, higher DMA content led to a reverse trend because of limited chain mobility. Excessive hydrogen bonds cause a reduction in chain mobility.^11^ Their results indicated that the optimum content of DMA depends on the balance between the adhesion strength and the flexibility of chains.

### Interaction with silica surface

**Fig. 8:**
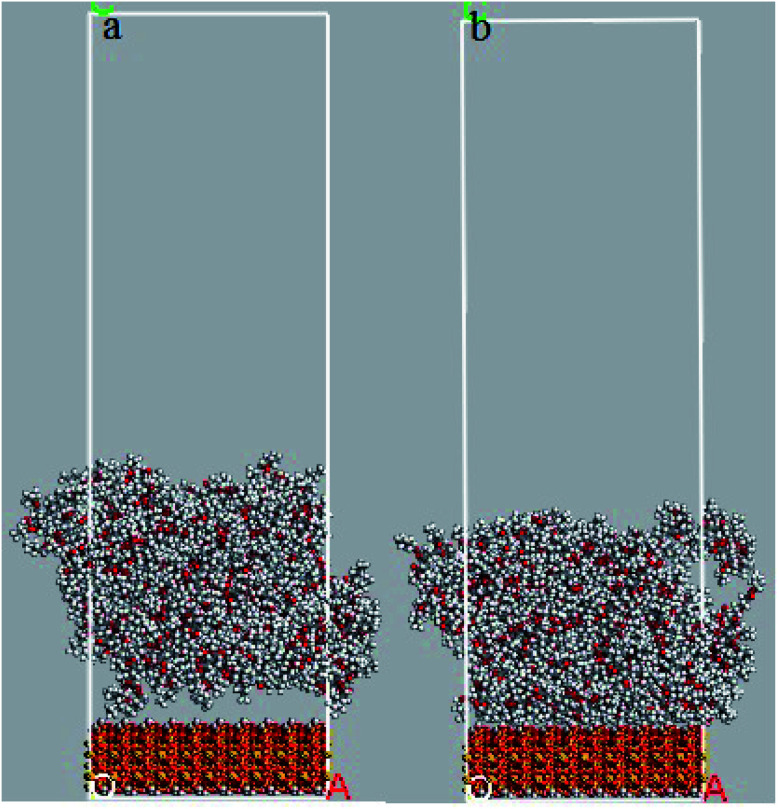
Snapshots of 36-4 copolymer on the silica layer (a) before equilibrium and (b) after equilibrium state.

In order to evaluate the effects of DMA and PMA on adhesion behavior and arrangement of atoms on the silica surface, the relative concentration of atoms perpendicular to silica surface and the radial distribution function between atoms of both layers are presented and discussed in this section. The snapshots of 36-4 copolymer on the silica layer before and after equilibrium are shown in Fig. 8. Due to the similar trends and results, only the plots of 36-4 copolymer, DMA 33-4-3, and PMA 34-4-2 are presented and discussed.

The relative concentration (RC) of copolymer and terpolymers atoms perpendicular to the silica layer is:10
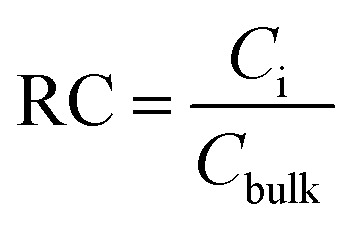
11
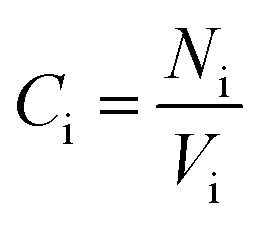
where *C*_i_, *C*_bulk_, *N*_i_, and *V*_i_ are the concentration of atom i in the slab, concentration of atom i in the bulk, number of i atoms in the slab, and volume of the slab, respectively.

**Fig. 9:**
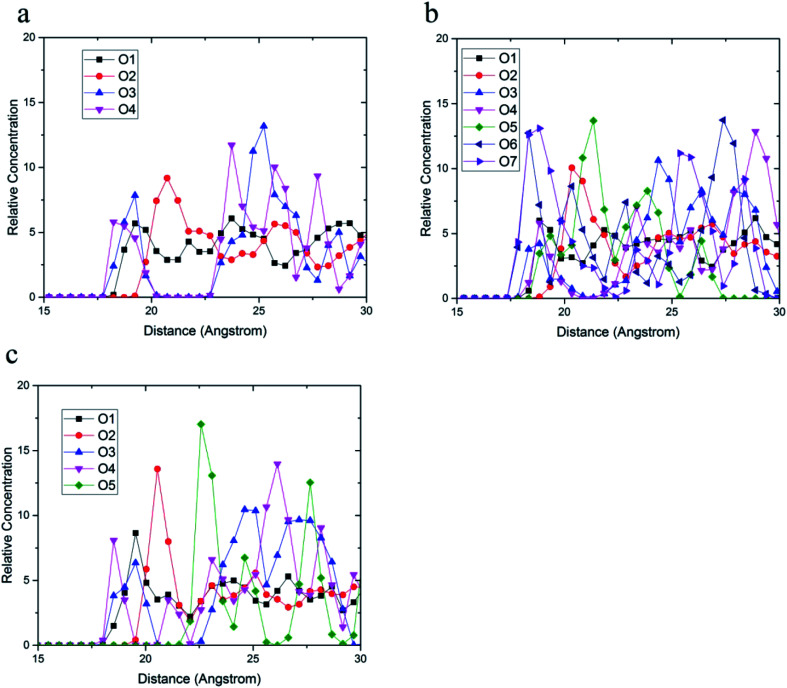
Relative concentration of oxygen atoms of (a) 36-4, (b) DMA 33-4-3, and (c) PMA 34-4-2 terpolymers.

The relative concentrations of oxygen atoms of 36-4 copolymer, DMA 33-4-3 terpolymer, and PMA 34-4-2 terpolymer are shown in Fig. 9.

In 36-4 copolymer, O3 and O4 oxygen atoms of acrylic acid comonomers were located at a lower distance from the silica surface compared to O1 and O2 oxygen atoms of butyl acrylate comonomers, which could be due to the slighter steric effect of O3 and O4 oxygen atoms compared to O1 and O2 atoms located in the side group of butyl acrylate comonomers. Furthermore, the steric effect of the butyl group on O2 was higher than O1 leading to a farther peak location in Fig. 9a. In DMA 33-4-3 terpolymer, the O5 oxygen atom located in the middle of the DMA side group was further away from the silica surface like O1 and O2 oxygen atoms as shown in Fig. 9b. However, O6 and O7 oxygen atoms, located at the end of the side group, could form hydrogen bonds with hydrogen atoms on the silica surface leading to the closest distance to the silica surface. According to Fig. 9c, there was no change in the relative concentration of O1, O2, and O3 oxygen atoms in PMA 34-4-2 terpolymer compared to 36-4 copolymer. However, O5 oxygen atoms showed the longest distance from the silica surface compared to other oxygen atoms of PMA 34-4-2 terpolymer.

Comparison of Fig. 9b and c reveals that unlike PMA comonomer, DMA caused the transfer of O3 and O4 peaks to a farther distance from the silica surface. It means that the role of acrylic acid comonomer was reduced by DMA comonomer, creating a gap and distance between the acrylic acid comonomer of the chains and the silica surface. In other words, terpolymer chains approaching the silica surface from the catechol side of DMA reduced the interaction of acrylic acid comonomer with the silica layer by the large side group of DMA. Payra *et al.*^11^ concluded that poly(alkyl methacrylate-dopamine methacrylate) copolymer could be oriented on metal and glass substrate by measuring lap-shear strength. They stated that copolymer chains oriented on the surface and approached the substrate from the catechol groups of DMA comonomer. The results of the simulation in the present study confirmed their statements and assumptions.

**Fig. 10:**
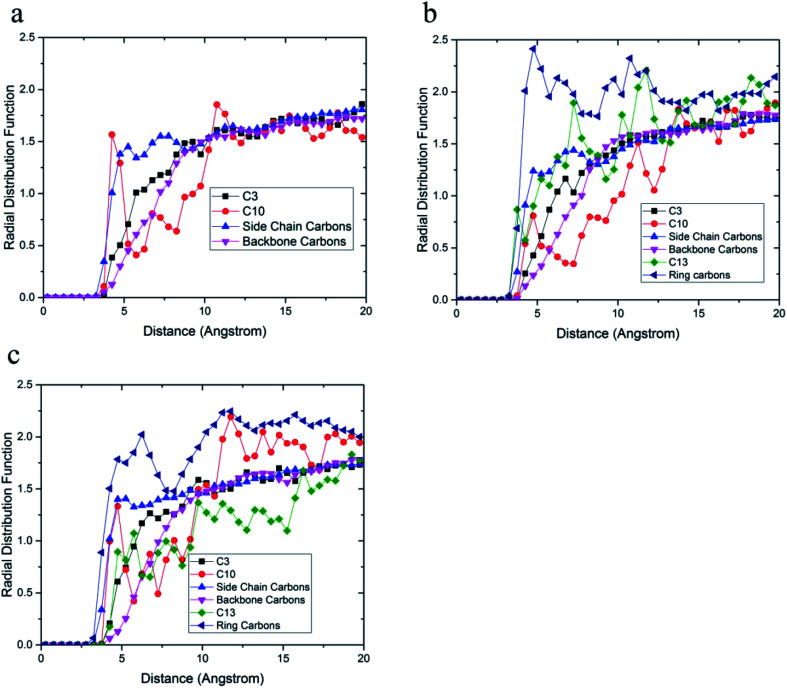
Radial distribution function between carbon atoms and topmost silica atoms (a) 36-4, (b) DMA 33-4-3, and (c) PMA 34-4-2.

The radial distribution function between carbon atoms and topmost silica atoms is shown in Fig. 10. Since the silica layer was considered as a fixed layer, higher peaks in the radial distribution function indicated more affinity to the silica surface like relative concentration plots.^51^ According to Fig. 10a, the peak of C10 carbons of the acrylic acid comonomer of the 36-4 copolymer was located at the closest distance from the silica surface followed by the peak of side group carbons of the butyl acrylate comonomer, *i.e.*, *C*_4_ + *C*_5_ + *C*_6_ + *C*_7_. Subsequently, the C3 carbon atom of the side group of butyl acrylate and the main chain carbon atoms *i.e. C*_1_ + *C*_2_ + *C*_8_ + *C*_9_ had the farthest distances from the surface silica atoms.

As shown in Fig. 10b, the highest peak in DMA 33-4-3 terpolymer was related to carbon atoms of the benzene ring of DMA comonomer, indicating that the terpolymer chain approached the silica surface from the catechol side. C13 of DMA comonomer, side chain carbon of butyl acrylate comonomer (*i.e. C*_4_ + *C*_5_ + *C*_6_ + *C*_7_), C10 carbon atom of acrylic acid comonomer, C3 carbon atom of butyl acrylate comonomer and the main chain carbon atoms *i.e. C*_1_ + *C*_2_ + *C*_8_ + *C*_9_ + *C*_11_ + *C*_12_ were farther from the silica surface in the order mentioned.

As shown in Fig. 10c, the highest peak was for the carbon atoms of the benzene ring in PMA 32-4-2 terpolymer. However, the difference between the peaks of the benzene ring and other carbons was reduced compared to DMA 33-4-3 terpolymer, indicating the main role of hydroxyl groups on the benzene ring of DMA comonomer in affinity to the silica surface. The side chain carbon atoms, *i.e. C*_4_ + *C*_5_ + *C*_6_ + *C*_7_ of butyl acrylate comonomer and C10 carbon atoms of acrylic acid comonomer were located after carbons of the benzene ring. In addition, the main chain carbon atoms, *i.e. C*_1_ + *C*_2_ + *C*_8_ + *C*_9_ + *C*_11_ + *C*_12_, showed the farthest radial distribution function due to the steric effects of the side groups.

The interaction energy between the silica and the polymer layer, *i.e. E*_int_, can be calculated using the following equation:

12 *E*_int_ = *E*_total_ − (*E*_polymer_ + *E*_silica_)

where *E*_total_, *E*_polymer_, and *E*_silica_ are the total energy of the system, energy of the polymer layer, and energy of the silica layer, respectively.

Adhesion energy is equal to the negative sign of the interaction energy. According to Table 4, the changes in interaction energies of terpolymers containing DMA and PMA comonomers were not noticeable compared to 36-4 copolymer. Therefore, in comparison to Fig. 3a, it can be concluded that PMA and DMA comonomers improved the cohesive energy of poly(*n*-butyl acrylate-*co*-acrylic acid); however, their effect on adhesion energy was not significant on the silica surface in the dry condition.

**Table 4:** Interaction energies between silica layer and copolymer and terpolymers

Name	Interaction energy (kcal mol^−1^)
36-4	−468.29
DMA 35-4-1	−471.54
DMA 34-4-2	−482.31
DMA 33-4-3	−466.19
PMA 34-4-2	−472.10

## Conclusion

The influence of DMA and PMA comonomers on the cohesive energy density, hydrogen bond formation and mechanical properties of the poly(*n*-butyl acrylate-*co*-acrylic acid) copolymer was investigated using molecular dynamics simulation. The increase in gyration radius caused by PMA comonomer was more than the increase due to DMA since intramolecular hydrogen bonds reduced chain expansion. The results demonstrated that DMA comonomer significantly enhanced the ductility index from a negative value to a positive value leading to behavior change from brittle to tough behavior confirmed experimental observation in literature. Moreover, the effect of PMA comonomer on improving the ductility index was less than the effect of DMA indicating a direct relationship with the electrostatic component of cohesive energy density. The presence of 12.2% by weight of DMA comonomer led to a 3.5-fold increase in the number of hydrogen bonds in the simulation box. The relative concentration perpendicular to the silica layer and the radial distribution function between topmost silica atoms and carbons of terpolymers indicated that the chains approached the silica layer from the benzene ring of DMA and PMA comonomers.

## Conflicts of interest

There are no conflicts to declare.

## Supplementary Material
